# A Case of Anti-Leucine-Rich Glioma-Inactivated Protein 1 (Anti-LGI1) Limbic Encephalitis With New-Onset Panic Attacks

**DOI:** 10.7759/cureus.58406

**Published:** 2024-04-16

**Authors:** Bre'Ana Coleman, Kiranpreet Sawhney, Paul LaPenna

**Affiliations:** 1 Neurology, Edward Via College of Osteopathic Medicine, Spartanburg, USA; 2 Neurology, Bon Secours St. Francis Downtown, Greenville, USA

**Keywords:** faciobrachial dystonic seizures (fbds), autoimmune encephalitis, autoimmune neurologic disorder, panic disorder, anti-lgi1 limbic encephalitis

## Abstract

Anti-leucine-rich glioma-inactivated protein 1 (anti-LGI1) limbic encephalitis is a rare autoimmune neurologic disorder with antibodies against LGI1. It was first recognized as a disease in 2010 and represents the second most common cause of autoimmune encephalitis. Clinically, it is characterized by subacute changes in cognition, memory, and behavior, associated with hyponatremia and faciobrachial dystonic seizures (FBDS).

This report discusses a unique onset of anti-LGI1 limbic encephalitis where an elderly female presented with symptoms of new-onset panic attacks and rhythmic facial movements for one week. She was then admitted to neurology for further serum, cerebrospinal fluid(CSF), and lab testing. She was eventually found to be positive for antibodies against LGI1 voltage-gated potassium channels, which confirmed the diagnosis of limbic encephalitis. The quick recognition of symptoms and escalation of management allowed the patient to experience drastic improvements after the initiation of steroids, immunotherapy, and lacosamide. Since anti-LGI1 limbic encephalitis is underdiagnosed, it can lead to irreversible long-term cognitive sequelae (i.e., memory deficits). Thus, awareness of the typically associated findings of FBDS, cognitive disturbances, psychiatric changes, and hyponatremia can aid in early diagnosis and prompt treatment with immunotherapy, allowing for more favorable outcomes.

## Introduction

Autoimmune limbic encephalitis (ALE) is a rare neurological disorder in which the immune system attacks proteins of the limbic system, resulting in inflammation of the brain [1]. ALE can be divided into paraneoplastic and non-paraneoplastic. Of the two, non-paraneoplastic ALE is more common and has a better prognosis due to its responsiveness to immunosuppressant drugs.

One of the proteins found to cause limbic encephalitis is the leucine-rich glioma-inactivated protein 1 (LGI1) [2]. Anti-LGI1 antibodies were first recognized as a disease in 2010 and represent the second most common cause of autoimmune encephalitis. Autoimmune anti-LGI1 limbic encephalitis generally affects older males but can occur between the ages of 30 and 80 years. Recent studies have indicated that anti-LGI1 limbic encephalitis has an annual incidence rate of 0.63-0.83 per one million and accounts for 11.2% of all autoimmune encephalitis [3].

LGI1 is a subunit of voltage-gated potassium channel (VGKC)-complex antibodies found predominantly in presynaptic and postsynaptic terminals of temporal lobe nerve cells [4]. When antibodies form against LGI1, it affects the brain's limbic system, resulting in acute to subacute neuropsychiatric changes. A characteristic symptom noted in 60% of patients is short-lived unilateral contractions of the arm and face, known as faciobrachial dystonic seizures (FBDS) [5]. These seizures can precede or occur concurrently with psychiatric and cognitive disturbances. Behavioral symptoms such as hallucinations or changes in emotional state have been noted as the initial presentation in 18-62.5% of patients. More commonly, however, they coexist with either memory changes or seizures. Cognitive changes in memory, spatial orientation, and mood have been seen as a primary manifestation of this disorder [3].

Diagnostic workup for anti-LGI1 limbic encephalitis includes serum labs, cerebrospinal fluid (CSF) studies, electroencephalogram (EEG), and brain imaging [4]. Antibody testing can be done in the serum and CSF; however, the serum is more sensitive. The CSF studies are typically normal with little to no inflammation in the CSF [6]. Additionally, due to the secretion of the LGI1 protein in the hypothalamus, a common associated finding on serum chemistry is hyponatremia [7]. EEG is abnormal in 80% of patients, revealing epileptic seizures or focal or diffuse slowing [5]. Although brain magnetic resonance imaging (MRI) can show normal results, it typically demonstrates hyperintensity in the mesial temporal lobes, hippocampi, and basal ganglia on the T2 fluid-attenuated inversion recovery (FLAIR) [2,4-5]. Underlying tumors are rare in LGI1 antibody-associated syndromes; nonetheless, it is important to rule out thymomas and lung cancer through computed tomography (CT) [5].

The purpose of this report is to discuss a case of anti-LGI1 limbic encephalitis in an elderly female with one week of new-onset panic attacks and rhythmic facial movements. Anti-LGI1 limbic encephalitis is underdiagnosed; hence, identifying the commonly co-occurring tetrad of cognitive disturbances, FBDS, psychiatric changes, and hyponatremia, will allow for a high clinical suspicion and prompt neuronal antibody testing to confirm the diagnosis and initiate treatment with immunotherapy [5,8].

## Case presentation

A 72-year-old Asian female with a history of controlled hypertension and hyperlipidemia presented to the emergency department with a chief complaint of sudden-onset panic attacks. This occurred for one week with no prior history of mood disorder. Her symptoms started with some vertigo and feeling off-balanced with occasional episodes of vomiting. The patient also noted a slight frontal headache and moments of disorientation to time and place. In addition, she noted feeling very anxious before experiencing repetitive rhythmic movements in her jaw that lasted about 20 seconds in duration before stopping. She was seen by her primary care provider at the onset of symptomology and was prescribed meclizine; however, there was no resolution.

The patient denied fever, recent illness, focal numbness, or weakness. She did not experience recent head trauma, chest pain, or abdominal pain. She denied a history of strokes nor cardiovascular illness. Other pertinent positive reviews of systems included fatigue, blurry vision, tremor, and dysarthria. The patient did not have a known family history or surgical history. Otherwise, she had no remarkable past medical history.

On neurological exam, the patient had poor comprehension and short-term memory; however, it was difficult to accurately assess due to the language barrier. Her language and speech were normal in her native language. The cranial nerve and motor exams were normal. Her bilateral patella muscle reflexes were absent. Her plantar response was flexor bilaterally. Sensation to light touch, pinprick, vibration, and proprioception were intact in all extremities. Her cerebellar examination was also normal. Multiple episodes of rhythmic movements of the facial musculature were seen on observation and associated with sudden-onset panic. These episodes lasted approximately 20-25 seconds, and each episode was identical.

Her basic metabolic panel shown in Table 1 demonstrated hypochloremia, hypokalemia, and hyponatremia with a decreased serum osmolality.

**Table 1:** Patient's basic metabolic panel upon hospital admission. Na: sodium; K: potassium; Cl: chloride

Basic metabolic panel	Values	Reference range
Na	120 mEq/L	135-145 mEq/L
K	3.4 mEq/L	3.5-5.5 mEq/L
Cl	86 mEq/L	101-110 mEq/L
Serum osmolality	249 mEq/L	275-295 mEq/L

The hepatitis B panel revealed positive surface antibodies, non-reactive surface antigens, and positive core total antibodies, indicating immunity from prior natural hepatitis B infection. She had no abnormalities in hematological examinations, cardiac lipids, thyroid-stimulating hormone (TSH), cortisol, or GI-liver profiles.

**Figure 1:**
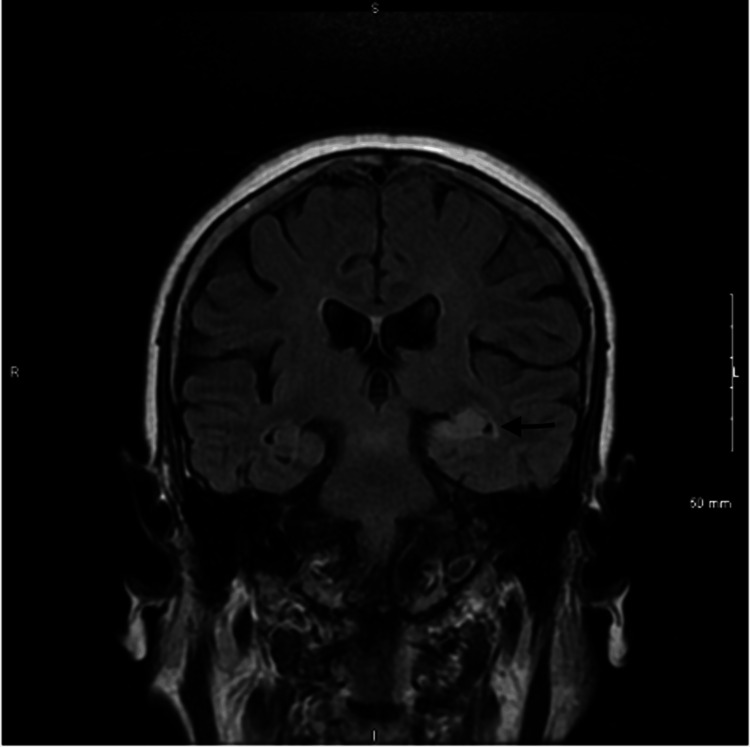
This coronal view of brain MRI FLAIR with no contrast shows an increased T2 signal that involves the left hippocampus and extends posteriorly. MRI: magnetic resonance imaging; FLAIR: fluid-attenuated inversion recovery

**Figure 2:**
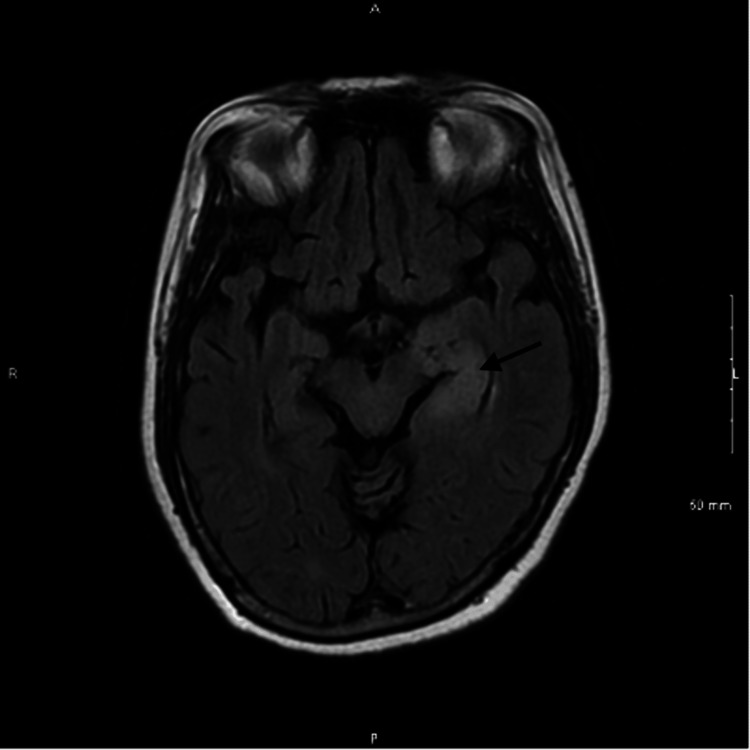
This axial view of brain MRI FLAIR with no contrast shows an increased T2 signal involving the medial left temporal lobe, which is consistent with limbic encephalitis. MRI: magnetic resonance imaging; FLAIR: fluid-attenuated inversion recovery

The patient's CT scan of the head without contrast revealed right maxillary mucosal thickening. The brain MRI with and without contrast demonstrated an abnormal increased T2 signal involving the medial left temporal lobe extending posteriorly (Figure 1, Figure 2), which is concerning for possible herpetic encephalitis versus low-grade glioma per radiology.

The EEG noted electrographic seizures arising from the left temporal lobe on at least four occasions lasting approximately 30 seconds in duration. Between electrographic seizures, periodic lateralized discharges were seen in the left hemisphere. Epileptiform discharges were also seen in the left temporal region. These findings correlated with her clinical rhythmic facial movements and acute-onset panic. Testing from the CSF, including cell count, protein, glucose, and meningitis panel (including testing for herpes simplex virus (HSV) 1 and 2), were all normal.

Given these discoveries, the patient's current syndrome was classic for anti-LGI1 limbic encephalitis. She was then started on lacosamide, steroids, and intravenous immunoglobulin (IVIG). She was also given acyclovir until herpes encephalitis could be ruled out. Throughout her five-day hospital course, she was placed on the treatment regimen of IV Solu-Medrol 1000 mg daily and IVIG (Gamunex-C, Grifols) 30 g daily for five days. Lacosamide 200 mg twice a day (BID) was also administered. Her hyponatremia was managed with urea-Na supplementation by nephrology. A CT of the chest, abdomen, and pelvis was ordered due to this specific syndrome's association with cancers such as small-cell lung cancer and thymomas. Her CSF and serum studies were sent out to the Mayo Clinic Laboratories for antibody testing.

The patient noted remarkable improvement within 12 hours of initiating antiepileptics and steroids. She denied further panic attacks or seizures. Herpes encephalitis was also ruled out and acyclovir was discontinued. Her active problems of hyponatremia, vertigo, hypokalemia, and focal seizures resolved. The patient continually improved to her neurologic baseline and was discharged on day 5 of hospitalization. She was instructed to follow up in the neurology clinic to discuss laboratory results and further treatment options. Fourteen days post-hospitalization marked a significant breakthrough as her serum results confirmed the presence of anti-LGI1 antibodies, providing crucial insight into her condition and advancing our diagnostic journey. Despite negative findings in her CSF studies, she was promptly scheduled for rituximab infusions as part of her outpatient management plan for targeted therapeutic intervention.

## Discussion

LGI1 is a neuronal protein found in high amounts in the hippocampus [9]. Antibodies formed against this region produce subacute changes in mood, memory, and cognition and present with the development of tonic seizures and hyponatremia. Studies have discovered that LGI1 functions as a ligand for ADAM22 and ADAM23, two epilepsy-related proteins [5,10]. Some patients experience tonic seizures due to the LGI1 antibodies blocking the LGI1-ADAM22 interaction [11]. FBDS are highly specific for anti-LGI1 encephalitis and are seen in about 60% of cases [12]. Tumors are rare in anti-LGI1 encephalitis, but when discovered, it is usually a thymoma. A CT of the chest, abdomen, and pelvis can help rule out neoplasm.

In the case reported, our patient presented with a chief complaint of new-onset panic attacks. With further investigation, it was discovered that her unprovoked symptoms of panic and fear derived from seizure activity in the amygdala. The amygdala is a temporal lobe structure that is mostly known for its role in emotions and is a key player in epileptogenesis and epilepsy [12]. In addition, her FBDS, MRI, and EEG findings were highly suggestive of anti-LGI1 encephalitis, which fueled the study of CSF and serum antibodies. Fourteen days later, the serum antibody panel concluded a positive presence of LGI1, confirming the clinical suspicion. Although CSF studies did not reveal the presence of antibodies, research has indicated that serum testing is more sensitive in detecting them.

The prognosis of anti-LGI1 limbic encephalitis is considered good with a low mortality rate [13]. Most patients regain their baseline neurologic function when diagnosed and treated promptly, as the patient did in the case presented. Yet, it is important to note that memory and cognitive complications can persist. Additionally, patients undergoing treatment with rituximab infusions may experience acute side effects such as infusion-related reactions of urticaria, tremor, fever, and tachycardia [14]. Twenty-five percent of patients may also experience disease flare-ups within the first couple of months due to treatment withdrawal. A study suggested that early and short-term rituximab therapy is presumptively a safe and effective treatment option for most patients with anti-LGI1 encephalitis [14].

## Conclusions

Anti-LGI1 encephalitis remains underdiagnosed, and high levels of suspicion are required for confirmation. It is important that clinicians consider autoimmune encephalitis in patients who present with subacute changes in cognition, memory, behavior, and, as recognized in this case, panic attacks. While CSF studies and brain imaging are useful tools, neuronal antibodies found in the CSF and serum are important for confirmatory testing and timely treatment initiation. Anti-LGI1 limbic encephalitis has favorable outcomes when treated early; thus, recognition of clinical and serological indicators is critical for preserved neurological function.
